# Supplementary material for: Ixeris polycephala Extract Alleviates Progression of Benign Prostatic Hyperplasia via Modification of Proliferation, Apoptosis, and Inflammation
Source: Pharmaceuticals (Basel). 2024 Aug 5;17(8):1032. doi: 10.3390/ph17081032 (PMC11357589; doi:10.3390/ph17081032)
Supplement: Supplementary file 1 [file pharmaceuticals-17-01032-s001.zip › pharmaceuticals-3116436-supplementary.pdf]

Supplementary material 1-1.

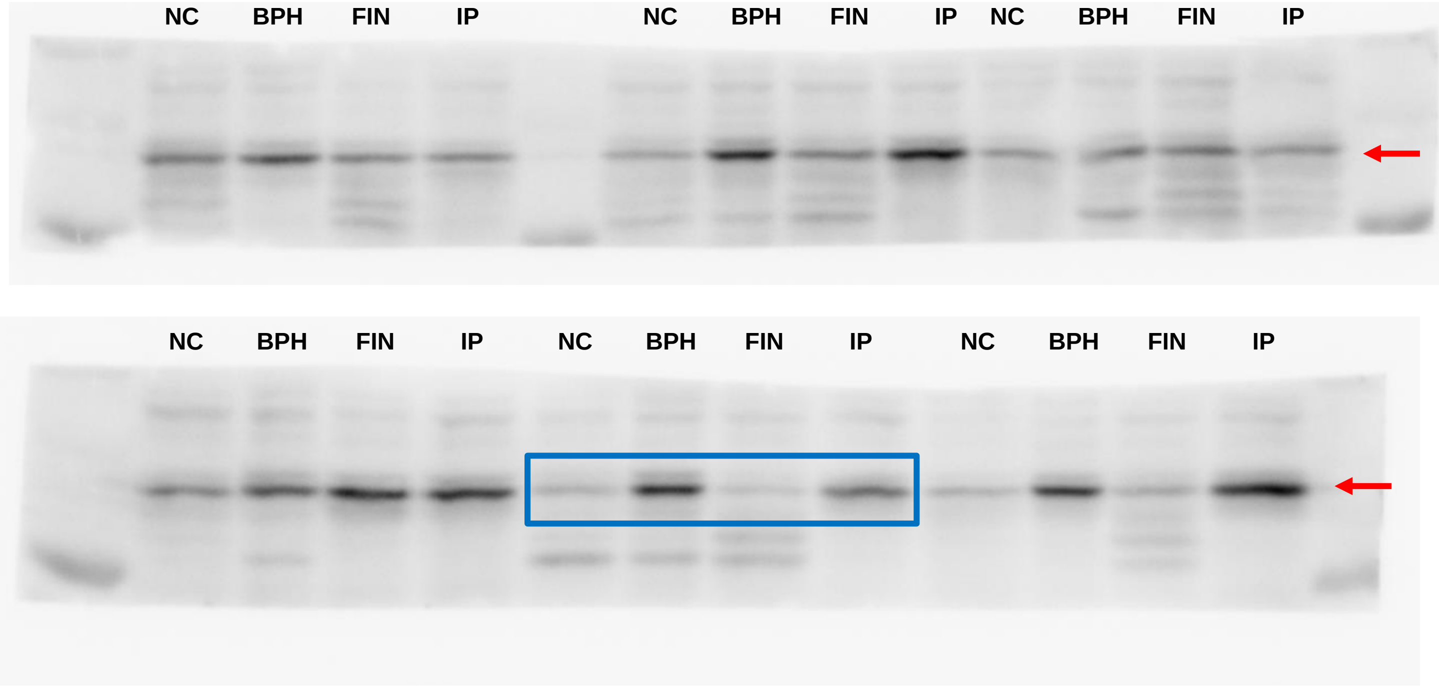

The Uncropped Blot of Cyclin D1 (36 kDa). The red arrow indicates the location of target bands. Blue box indicates the cropped region of Fig.4C

Supplementary material 1-2.

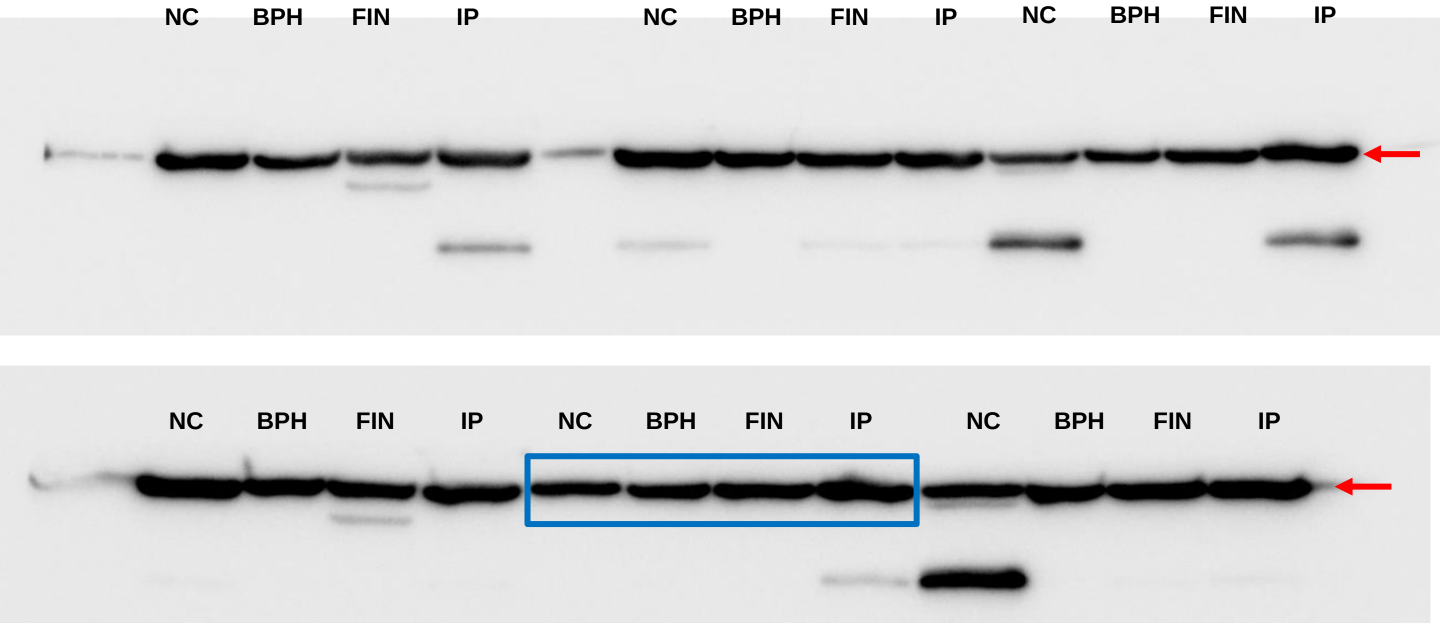

The Uncropped Blot of β-actin (42 kDa). The red arrow indicates the location of target bands. Blue box indicates the cropped region of Fig.4C

Supplementary material 1-3.

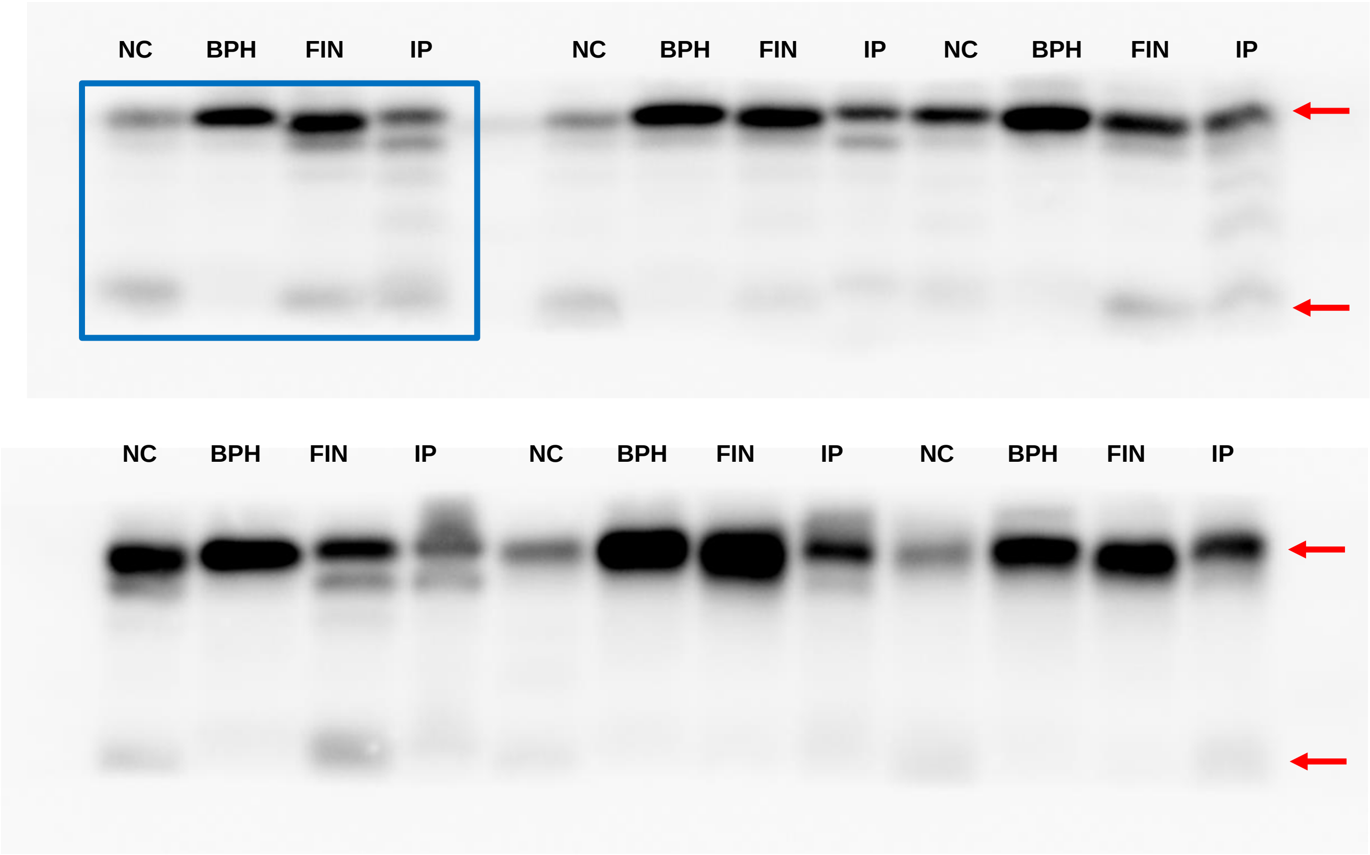

The Uncropped Blot of caspase-3 (17, 35 kDa). The red arrow indicates the location of target bands. Blue box indicates the cropped region of Fig.5A

Supplementary material 1-4.

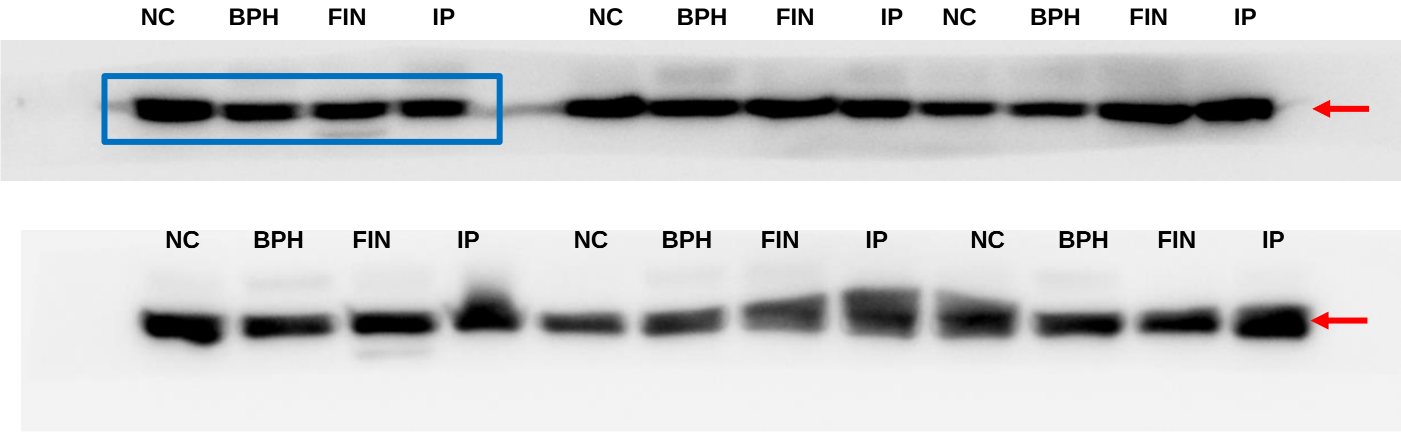

The Uncropped Blot of β-actin (42 kDa). The red arrow indicates the location of target bands. Blue box indicates the cropped region of Fig. 5A

Supplementary material 1-5.

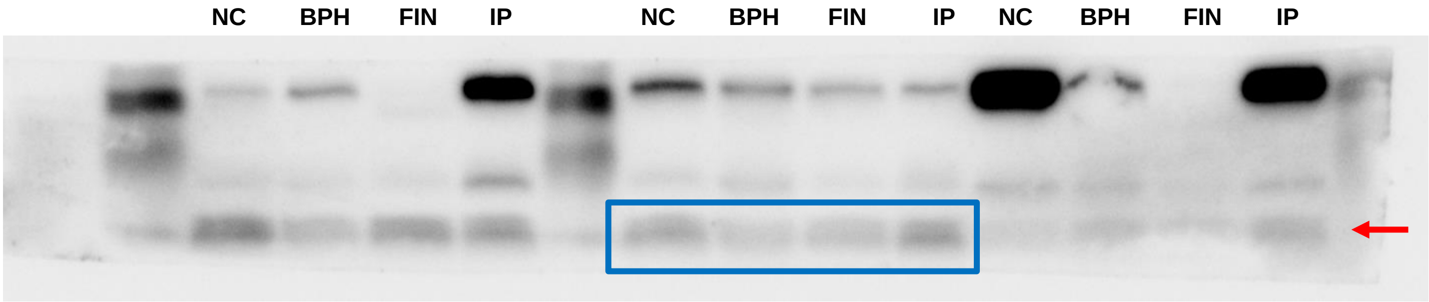

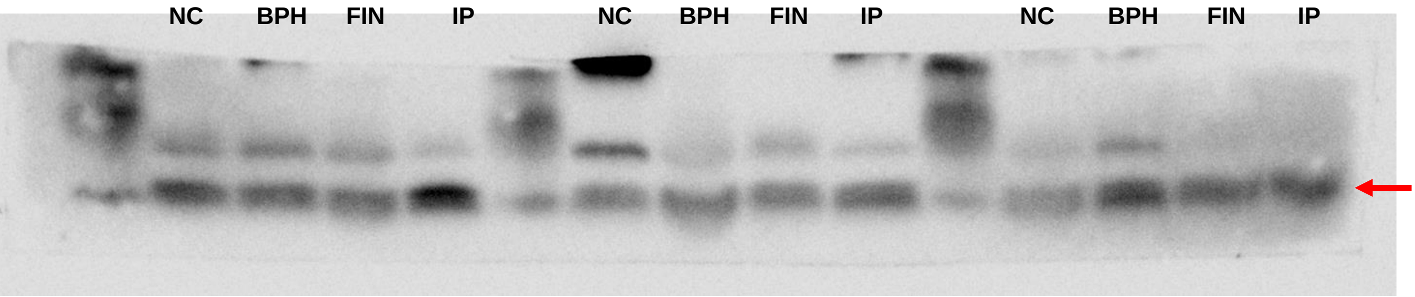

The Uncropped Blot of Bax (23 kDa). The red arrow indicates the location of target bands. Blue box indicates the cropped region of Fig.5B

Supplementary material 1-6.

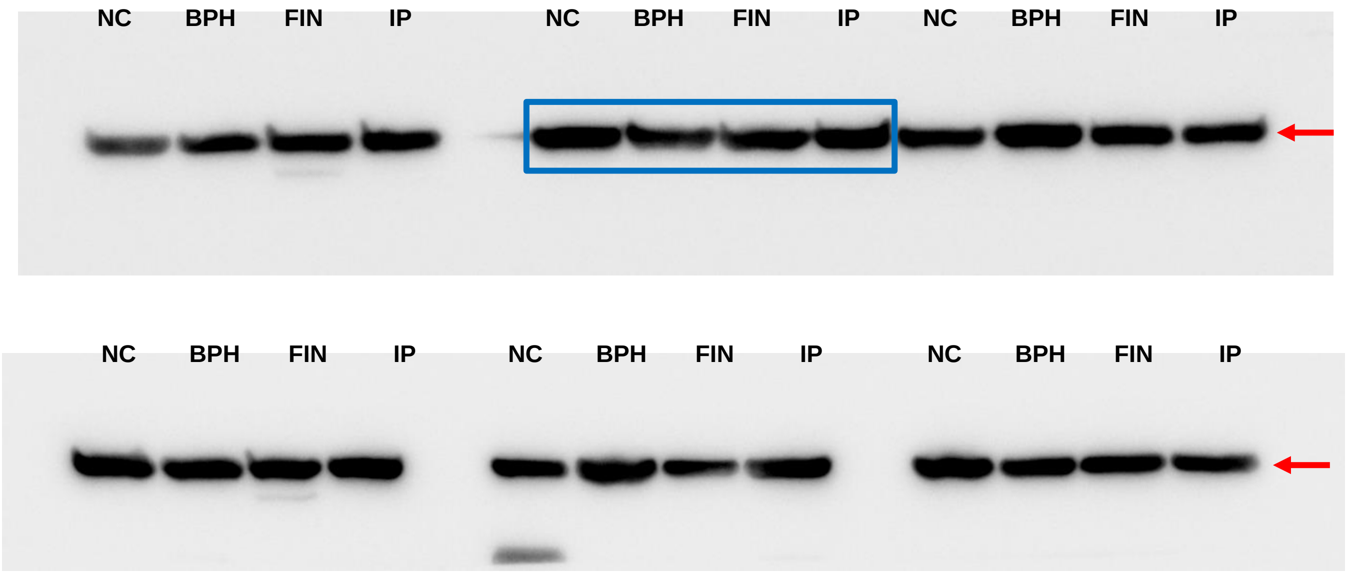

The Uncropped Blot of β-actin (42 kDa). The red arrow indicates the location of target bands. Blue box indicates the cropped region of Fig.5B

Supplementary material 1-7.

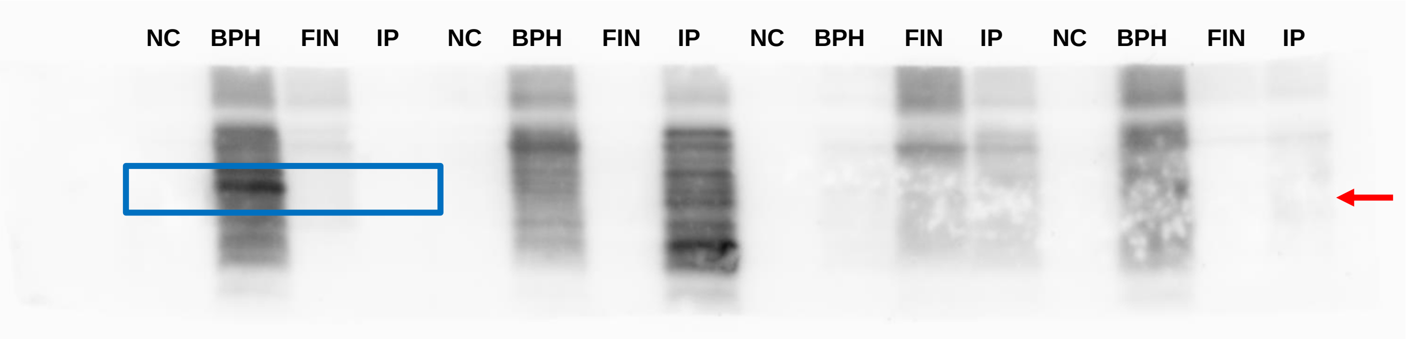

The Uncropped Blot of Bcl-2 (26 kDa). The red arrow indicates the location of target bands. Blue box indicates the cropped region of Fig. 5C

Supplementary material 1-8.

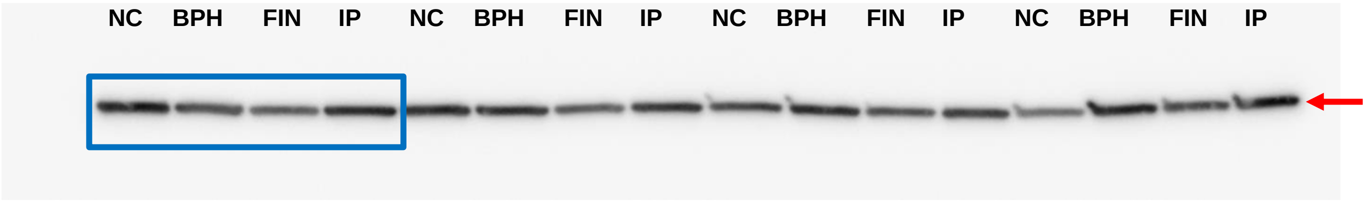

The Uncropped Blot of β-actin (42 kDa). The red arrow indicates the location of target bands. Blue box indicates the cropped region of Fig.5C

Supplementary material 1-9.

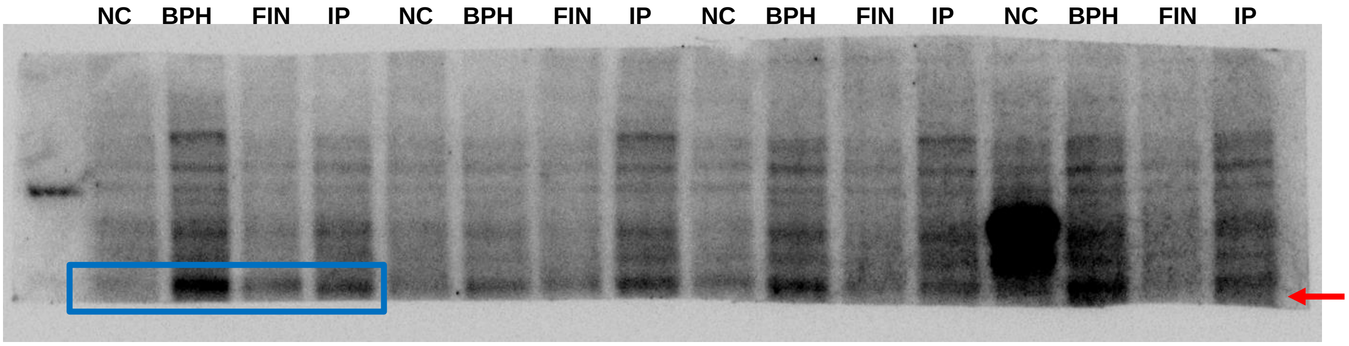

The Uncropped Blot of COX-2 (74 kDa). The red arrow indicates the Location of target bands. Blue box indicates the cropped region of Fig. 7A

Supplementary material 1-10.

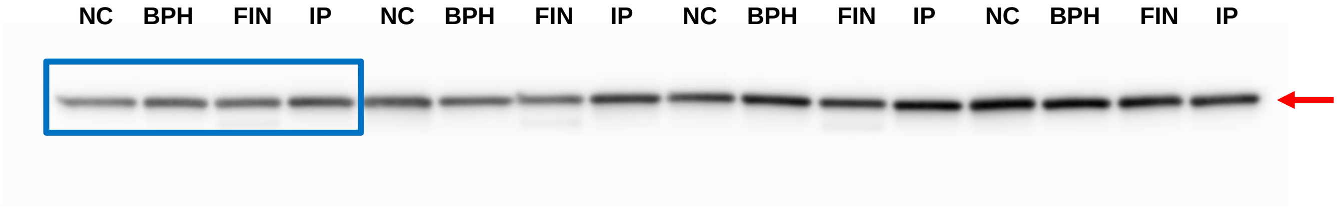

The Uncropped Blot of β-actin (42 kDa). The red arrow indicates the location of target bands. Blue box indicates the cropped region of Fig.7A

Supplementary material 1-11.

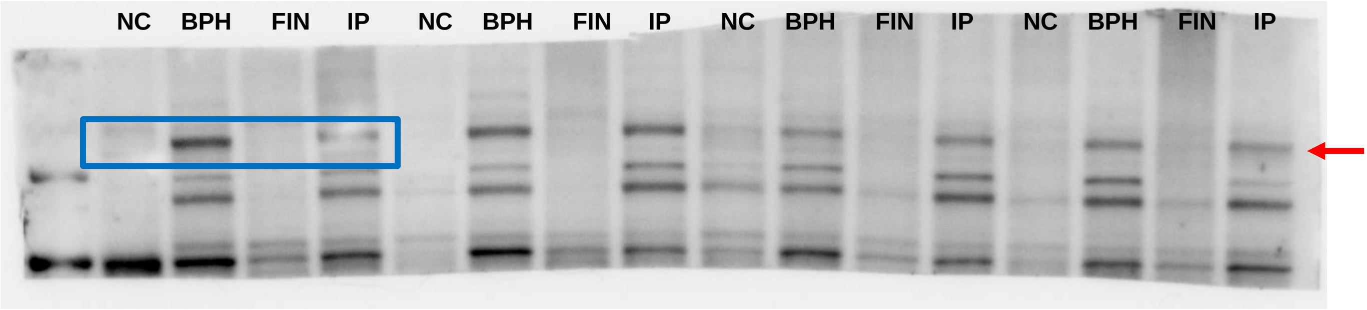

The Uncropped Blot of iNOS (130 kDa). The red arrow indicates the Location of target bands. Blue box indicates the cropped region of Fig. 7B

Supplementary material 1-12.

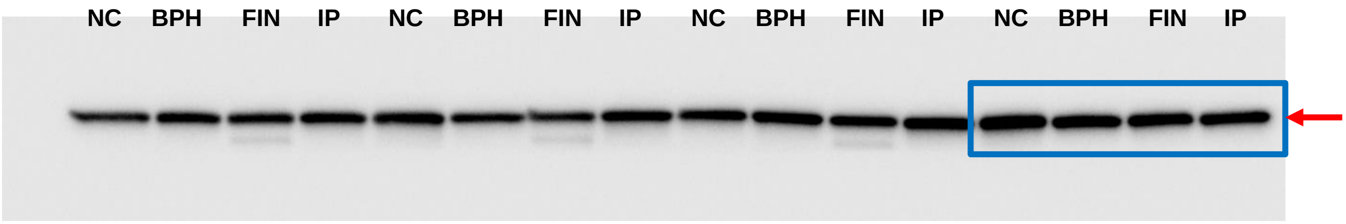

The Uncropped Blot of β-actin (42 kDa). The red arrow indicates the location of target bands. Blue box indicates the cropped region of Fig.7B
